# Button-Type Beam Position Monitor Development for Fourth-Generation Synchrotron Light Sources: Numerical Modeling and Test Bench Measurements

**DOI:** 10.3390/s24092726

**Published:** 2024-04-25

**Authors:** Stefano Cleva, Silvano Bassanese, Massimiliano Comisso, Moussa El Ajjouri, Rudi Sergo, Christian Morello, Andrea Passarelli

**Affiliations:** 1 Elettra Sincrotrone Trieste, Area Science Park, 34149 Trieste, Italy; 2 Department of Engineering and Architecture, University of Trieste, Via A. Valerio 10, 34127 Trieste, Italy; 3 National Inter-University Consortium for Telecommunications, Via S. Marta 3, 50139 Florence, Italy; 4 Synchrotron SOLEIL, L’Orme des Merisiers, 91190 Saint-Aubin, France; 5 National Institute for Nuclear Physics, Naples Unit, Via Cintia, 80126 Naples, Italy

**Keywords:** beam position monitor, diffraction-limited light source, storage ring, wakefield, electromagnetic simulation, test bench measurements

## Abstract

This paper addresses the design of beam position monitor (BPM) devices suitable for fourth-generation diffraction-limited X-ray storage rings. Detailed investigations of the electromagnetic (EM) phenomena occurring inside the component under various working conditions are carried out by considering different BPM EM models defined by their geometry and materials. Moving from a theoretical characterization of the common round geometry, rhomboidal structures are studied through a careful numerical analysis relying on advanced computer-aided tools. Several critical elements, such as wakefields, pick-up signal extraction, and trapped and propagating modes, are explored from the simulation point of view and from the experimental one, by deploying a manufactured microwave test bench, which is employed to measure the radio frequency behavior of a BPM prototype built at Elettra Sincrotrone Trieste. The aim of the proposed study is to identify a satisfactory tradeoff between achievable performance and practical realizability for BPM devices operating in last-generation light sources.

## 1. Introduction

To detect the longitudinal and transverse positions of electron bunches running inside a storage ring, a number of beam position monitor (BPM) devices must be placed along the orbit of the beam [1,2,3]. The performance of these BPMs influences the beam control capability by significantly affecting the characteristics of the emitted photons, which, in turn, are related to the properties defined by the machine optics on the stored beams. As a rule of thumb, the design complexity of the BPMs increases over time, moving from one generation of light sources to the next [4,5,6], in order to meet the increasingly stringent design constraints required to satisfy the user expectations. Fourth-generation synchrotron light sources [7,8] optimized for X-ray production, compared to third-generation machines, have higher stored current, shorter bunch lengths, smaller vacuum pipe cross-sectional dimensions, and non-uniform filling patterns. All these elements require the adoption of computer-aided engineering tools for BPM design. By simulating the detailed behaviour of BPMs under different operating conditions, it is possible to assess the various electromagnetic (EM) and thermal effects that may have been overlooked or approximated by simplified analytical models in the past but are no longer negligible today.

The BPM, by its nature, is an integral part of the accelerator vacuum pipe that operates very close to the stored particles and its beam coupling effects have to be reduced as much as possible to ensure a low impact on the global machine impedance. A low beam coupling impedance contribution is also beneficial in terms of dissipated power (wake losses) and local heating effects. To detect the longitudinal and transverse positions of the charged particle bunches, part of the beam EM energy has to be extracted by the BPM device, thus introducing an additional loading effect that increases the BPM beam coupling impedance. All possible machine operating conditions drive towards extremely wide range conditions for the signals that have to be picked up by the BPMs. Low loading effects on the beam and high enough signals for the front-end electronics impose conflicting requirements on the BPMs design task. The extremely wide beam spectrum compared to the narrow portion used by the front-end, finally introduces further EM issues that have to be carefully considered in the BPM design.

To address these issues, this paper describes the BPM development for a fourth-generation X-ray light source by accurately investigating its EM behavior [9,10,11,12,13,14]. A theoretical model of the typical round shape structure is firstly illustrated to then move towards a detailed numerical EM characterization of the more sophisticated rhomboidal geometry. Different materials and operation conditions are explored in terms of wake potential and impedance, spectrum, and trapped and propagating modes to finally identify a suitable BPM design capable to combine a satisfactory performance with a manageable realizability [15,16]. The simulation results are completed by time domain reflectometer (TDR) and vector network analyzer (VNA) measurements, with the aim of checking the practical capabilities of a BPM prototype assembled at Elettra Sincrotrone Trieste.

The paper is organized as follows. Section 2 introduces the theoretical BPM model. Section 3 presents the numerical setup and discusses the simulation results. Section 4 describes the experimental testbed and illustrates the acquired measurements. Finally, Section 5 summarizes the main conclusions.

## 2. Basic Theoretical Analysis

**Figure 1:**
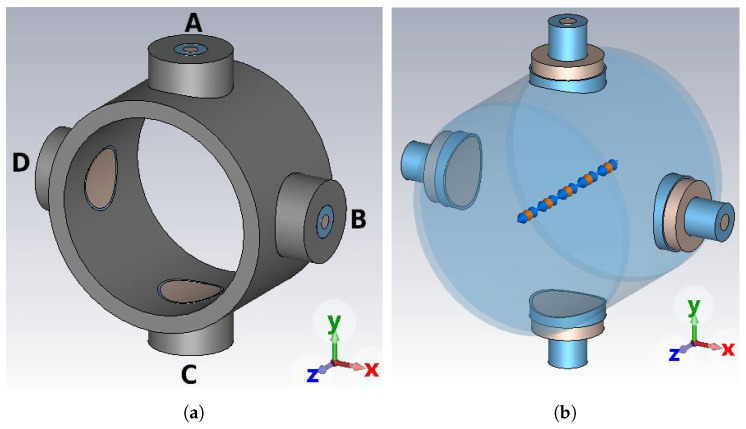
Round shape BPM structure: (**a**) body assembly, (**b**) EM model.

The typical button-type BPM with a vacuum pipe of circular-cross-section commonly used in particle accelerators is reported in Figure 1a. Four identical round electrodes, also known as electrostatic or capacitive coupled pick-ups (PUs), are located at azimuthally symmetric positions around the longitudinal *z*-axis of the device, corresponding to the symmetry axis of the cylindrical vacuum pipe.

When an ultra-relativistic axis-symmetric charged particle beam propagates along this axis (centered beam condition), the EM coupling between the beam field and the four PUs results the same for each electrode (Figure 1b). In this situation, four identical voltage signals are available at the end of each coaxial line that is feeded by its own PU. Sometimes, instead of the voltage, the power extracted from each coaxial port, usually designed to have a characteristic impedance of 50 Ω, is used as the beam induced quantity. In both cases, the signals delivered to the BPM ports A, B, C, and D constitute a balanced bridge. Otherwise, when the centered beam condition is not satisfied, the transverse beam position with respect to the *z*-axis may be evaluated by calculating the differences between the output voltages (or powers) by considering the (A, C) electrode pair for the *y* displacement and the (B, D) one for the *x* displacement.

**Figure 2:**
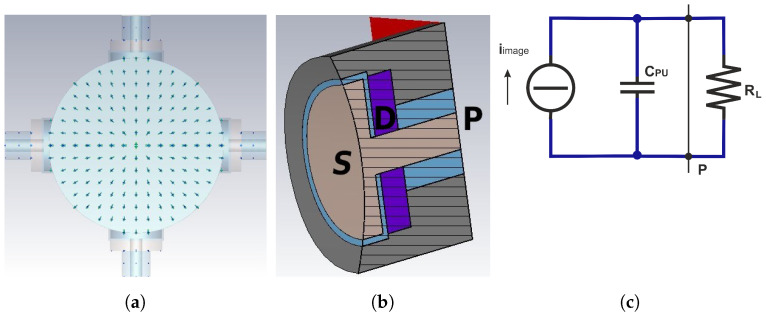
Round BPM model: (**a**) electric field, (**b**) PU cross-section, (**c**) equivalent circuit.

The characteristics of the signals delivered at the four ports can be inferred once the three-dimensional (3D) distribution of the EM field excited in the BPM volume is known. The usually adopted EM BPM model relies on some simplifying hypotheses. Firstly, the BPM body is assumed to be a cylindrically symmetric perfectly conducting structure of virtually infinite length. Secondly, the transverse distribution of the beam charge is assumed azimuthally symmetric with respect to the beam propagation direction, without imposing constraints on the longitudinal beam charge density. Thirdly, the on-axis beam condition is matched (in this paper only such kind of excitation is considered). When these three assumptions hold, a pure transverse EM (TEM) field propagates along the BPM, whose electric E and magnetic H field lines are, respectively, orthogonal (Figure 2a) and tangent to the inner surface of the vacuum pipe. This determines the induction, on the inner surface of the vacuum pipe, of an image charge and of a surface current both azimuthally symmetric but having polarity opposite to, respectively, the beam charge and the beam current. During the BPM manufacturing process, the real material is selected to have a relative magnetic permittivity of unity, while its finite electric conductivity is high enough that both the longitudinal electric field component introduced by the ohmic losses on the conducting surfaces and the skin depth penetration of the surface currents do not introduce significant deviations from the ideal TEM case.

### 2.1. Transfer Impedance and Signal Extraction

Since an opposite-polarity image charge is induced on the surface of each electrode illuminated by the beam (Figure 2a), the longitudinal surface charge density profile results proportional to the longitudinal charge profile of the particle beam by a coverage factor. The total induced charge $q_{\mathrm{image}}\left(t\right)$ as a function of the time *t* can then be calculated by integrating the surface charge density distribution over the electrode surface *S* (Figure 2b). This integration may be a very complex task that, if the bunch length is longer than the button radius (narrowband beam), can be approximately estimated assuming as uniform the longitudinal charge density of the beam over the button length. Under this condition, one obtains:

(1) $$q_{\mathrm{image}}\left(t\right)=\rho_{L}\left(z-ct\right)\;\frac{r_{\mathrm{PU}}^{2}}{2r_{\mathrm{pipe}}}=i_{\mathrm{beam}}\left(t\right)\;\frac{r_{\mathrm{PU}}^{2}}{2c\;r_{\mathrm{pipe}}},$$

where $\rho_{L}\left(z-ct\right)$ is the linear charge density of the exiting beam, $r_{\mathrm{PU}}$ is the button radius, $r_{\mathrm{pipe}}$ is the vacuum pipe radius, $i_{\mathrm{beam}}\left(t\right)$ is the beam current, and *c* is the speed of light in the vacuum. By taking the derivative of Equation (1), the current driven by the induced charge can be determined as:

(2) $$i_{\mathrm{image}}\left(t\right)=\frac{dq_{\mathrm{image}}\left(t\right)}{dt}=\frac{r_{\mathrm{PU}}^{2}}{2c\;r_{\mathrm{pipe}}}\;\frac{di_{\mathrm{beam}}\left(t\right)}{dt}.$$

If the signal extraction port P (Figure 2b) is left open, this current firstly charges and then discharges the button capacitance $C_{\mathrm{PU}}$, and thus the voltage can be calculated as:

(3) $$v_{\mathrm{port}}\left(t\right)=\frac{q_{\mathrm{image}}\left(t\right)}{C_{\mathrm{PU}}}=\frac{r_{\mathrm{PU}}^{2}}{2c\;r_{\mathrm{pipe}}C_{\mathrm{PU}}}\;i_{\mathrm{beam}}\left(t\right).$$

If P is instead terminated on an external load $R_{L}$, the equivalent current generator feeds the parallel connection of $C_{\mathrm{PU}}$ and $R_{L}$, hence producing a voltage drop across $R_{L}$ (Figure 2c). The evaluation of the impulse response corresponding to the $R_{L}-C_{\mathrm{PU}}$ parallel circuit completes the time domain analysis, since $v_{\mathrm{port}}\left(t\right)$ can be calculated by convolution once $i_{\mathrm{beam}}\left(t\right)$ is known. This enables to evaluate the PU transfer impedance as:

(4) $$Z_{T_{\mathrm{PU}}}\left(f\right)=\frac{V_{\mathrm{port}}\left(f\right)}{I_{\mathrm{beam}}\left(f\right)}=\frac{r_{\mathrm{PU}}^{2}}{2c\;r_{\mathrm{pipe}}C_{\mathrm{PU}}}\;\frac{j2\pi fR_{L}C_{\mathrm{PU}}}{1+j2\pi fR_{L}C_{\mathrm{PU}}},$$

where *f* is the frequency, $V_{\mathrm{port}}\left(f\right)$ and $I_{\mathrm{beam}}\left(f\right)$ are the Fourier transforms of the voltage drop and of the beam current, respectively, while $j=\sqrt{-1}$ is the imaginary unit. It is important to note that Equation (4) approaches a real constant in the high-frequency range ($f\gg f_{\mathrm{cut}}$) and an imaginary frequency-dependent function in the low-frequency one ($f\ll f_{\mathrm{cut}}$), with:

(5) $$f_{\mathrm{cut}}=\frac{1}{2\pi R_{L}C_{\mathrm{PU}}},$$

identifying the cutoff frequency. In the direct current (DC) case, that is, when f=0, the PU transfer impedance becomes equal to zero. This result matches the capacitive nature of the coupling between the beam and the electrode surface. Only bunch shapes that have a longitudinal profile that varies over time can be detected, as indicated by Equation (4). In practical applications, the analog front-end electronics connected to the four ports of each BPM are designed to operate at a specific centered frequency $f_{\mathrm{detect}}$ over a bandwidth Δf. To improve the resulting signal-to-noise ratio, the module of $Z_{T_{\mathrm{PU}}}\left(f_{\mathrm{detect}}\right)$ should be as high as possible. In this regard, by more deeply examining Equation (4), one may note that the parameters influencing this value are, on one hand, $r_{\mathrm{pipe}}$ and $R_{L}$, which are, however, determined by the design of other accelerator components, and, on the other hand, $r_{\mathrm{PU}}$ and $C_{\mathrm{PU}}$, which can instead be used to optimize the PU transfer impedance module.

If the maximum bunch spectrum frequency is low enough, as in the case of long beams, the total PU capacitance can be theoretically calculated by adding all the partial DC contributions due to the different sections that compose the PU itself. Accordingly, one can write:

(6) $$C_{\mathrm{PU}}=C_{\mathrm{gap}}+C_{\mathrm{transition}}+C_{\mathrm{coaxial}}+C_{\mathrm{fringe}},$$

where the four added capacitances $C_{\mathrm{gap}}$, $C_{\mathrm{transition}}$, $C_{\mathrm{coaxial}}$, and $C_{\mathrm{fringe}}$ refer, respectively, to the gap between the PU electrode and the housing, to the transition region between the same electrode and the coaxial line feeding port P, to the coaxial line itself, and to the fringe electric field. For simple electrode geometries, such as the cylindrical one used in Figure 2b, $C_{\mathrm{gap}}$ can be calculated by applying the formula for cylindrical capacitors when the button and gap sizes as well as the characteristics of the dielectric material in which the electric field is stored are all known. The same approach may adopted to derive $C_{\mathrm{coaxial}}$, while $C_{\mathrm{transition}}$, and even more $C_{\mathrm{fringe}}$, are difficult to estimate because of, respectively, non-uniform and dispersed electric field distributions inside the respective regions. As the bunch length decreases, the bunch spectrum width unavoidably increases and the lumped model that describes the transfer impedance becomes more and more inaccurate, to the point where the estimation of the PU capacitance becomes unfeasible. Due to the complex shape and the material characteristics of the PU, the field theory of guided waves has to be used for the computation of the signals available at the BPM ports. This approach is so complex that, apart from very simple coaxial geometries, the PU high-frequency behavior must be studied by numerical methods [17,18].

### 2.2. Longitudinal Coupling Impedance and Wake Losses

During the propagation along the BPM axis, the beam is affected by energy losses due to different EM coupling phenomena between the beam itself and its surroundings. Apart from the specific nature of the coupling mechanism, each type of loss can always be modeled by an impedance, that is, by a voltage drop along the beam path scaled to the beam current [19,20].

Due to the finite conductivity of real conductors, the surface currents induced by the beam give rise to ohmic losses that, in turn, introduce a longitudinal component of the electric field along the inner BPM surface. This effect, known as resistive wall, extends over the full beam frequency spectrum (wideband effect), with the corresponding energy loss that can be calculated by the flow of the Poynting vector through the inner BPM surface. The higher the conductivity of the BPM body, the lower the losses due to the resistive wall. As observed in the previous subsection, another factor contributing to the beam energy loss arises from the capacitive coupling. Here, the loading effect on the beam, caused by the extraction of a portion of its energy through each PU, serves as the necessary loss to generate the beam position signal. Assuming a narrow-band beam, the longitudinal beam coupling impedance of each PU is related to its respective transfer impedance by [21]:

(7) $$Z_{L_{\mathrm{PU}}}\left(f\right)=\frac{r_{\mathrm{PU}}^{2}}{2c\;r_{\mathrm{pipe}}R_{L}C_{\mathrm{PU}}}Z_{T_{\mathrm{PU}}}\left(f\right),$$

which hence shows the same frequency behavior of the PU transfer impedance. Further beam energy loss contributions arise from the high order modes (HOM) trapped in the gap that isolates the electrode surface from the vacuum pipe. Such HOMs act as narrowband resonators, whose quality factor should be kept as low as possible to reduce localized heating effects. In a real BPM, all the three mentioned losses and their respective impedances simultaneously coexist, with their relative impact varying according to the bunch spectrum. Ideally, a real BPM should limit the losses to only resistive wall and signal extraction contributions. The cumulative effect of all losses may be synthetically summarized by the loss factor:

(8) $$k_{∥}=\frac{\Delta U}{{\left(Q_{\mathrm{tot}}\right)}^{2}},$$

in which ΔU denotes the total energy lost by the beam passing through the BPM and $Q_{\mathrm{tot}}$ represents the total bunch charge. During the BPM design, $k_{∥}$ should be kept as low as possible, but this constraint could severely affect $Z_{T_{\mathrm{PU}}}$, which, ideally, should instead be as high as possible.

## 3. Numerical Analysis

As outlined in the previous section, the lumped nature of the analytical PU model limits its ability to describe the beam-PU coupling mechanism and the PU transfer function in a wideband context. To overcome this limitation, CST Particle Studio Suite [22] is adopted as a fully 3D numerical tool for modeling the EM field propagation inside the vacuum pipe and the PU.

The CST wakefield solver, in particular, is used to simulate the time domain (TD) EM behavior of a BPM device when excited by a relativistic beam. The simulation settings are selected as follows.

**Figure 3:**
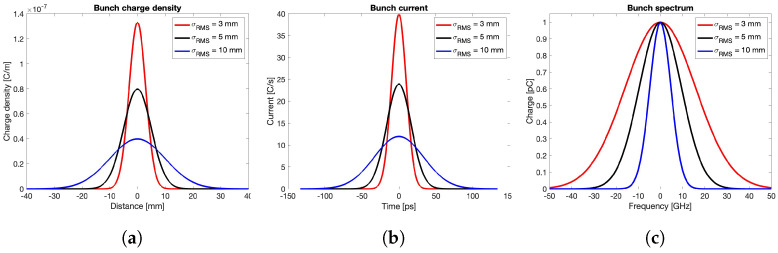
Gaussian bunches: (**a**) longitudinal charge density, (**b**) bunch current, (**c**) bunch spectrum.

- The bunch shape is represented by a Gaussian distribution characterized by a standard deviation $\sigma_{\mathrm{rms}}$, expressed in length unit (mm), and by a total charge $Q_{\mathrm{tot}}$, identified by the area under the curve. In the presented simulations, this latter parameter is fixed to 1 nC, while different $\sigma_{\mathrm{rms}}$ values ranging from 3 to 10 mm are considered. With reference to the shapes reported in Figure 3a, the vertical axis unit represents the linear charge density in C/m. Taking into account the propagation characteristics of the charged bunch at the speed of light, the horizontal axis can be rescaled in time unit (ns), and the vertical axis in electric current unit (C/s). The TD representation of the Gaussian distributions is shown in Figure 3b, while the corresponding spectra, obtained by the Fourier transformation, are reported in Figure 3c.
- Concerning the adopted materials, all of them are realistically characterized by a lossy behavior and a relative magnetic permeability $\mu_{r}=1$. In particular, stainless steel AISI316L with electric conductivity $\sigma_{\mathrm{body}}=1.35$ MS/m is chosen for the BPM body, while molybdenum with electric conductivity $\sigma_{\mathrm{button}}=18.2$ MS/m is selected for the central pin and the PU button. The vacuum sealing D (Figure 2b) is modeled by a dielectric material with relative electric permittivity $\varepsilon_{r}$, whose value ranges from 1 to 10.
- Regarding the simulator configuration, the beam input and output cross-sections of the vacuum pipe are set as open boundaries to emulate an infinitely long vacuum pipe, in order to avoid perturbations of the incoming and outgoing quantities. The signal propagating along the coaxial feedthrough of the PU is terminated on a 50 Ω matched waveguide port. Additionally, only one fourth of the BPM structure is required to be actually simulated by the CST wake solver, thanks to the on-axis beam excitation and the symmetry of the component. The mesh density is always set fine enough to properly cover the gap between the button and its housing.

According to these settings, the following subsections numerically investigate progressively more complex geometries by moving from the typical round BPM structure theoretically characterized in the previous section. In particular, the limitations of the basic analytical model are firstly addressed, while rhomboidal vacuum pipes are subsequently studied in comparison to the round chamber.

### 3.1. Round Pipe with Cylindrical PUs

**Figure 4:**
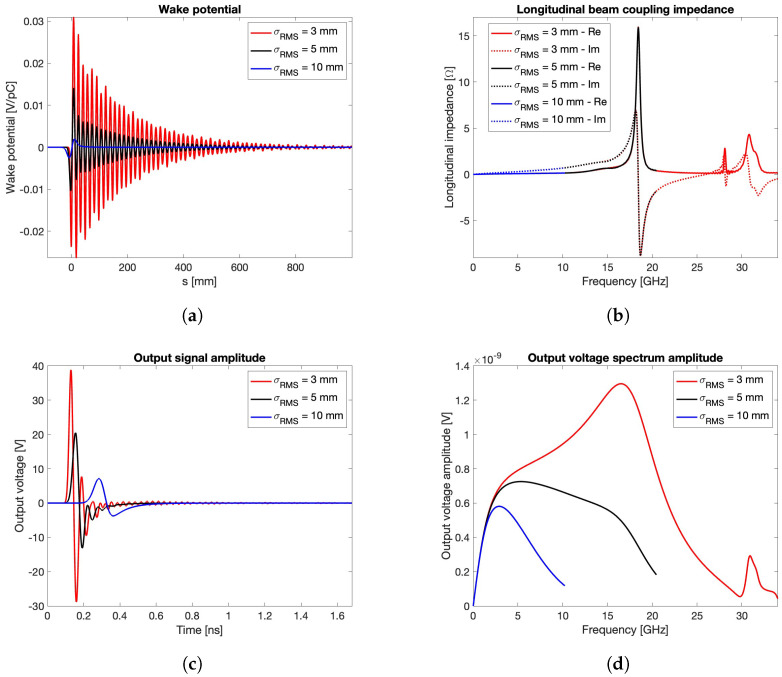
Wake potential (**a**), beam coupling impedance (**b**), output signal voltage (**c**), and spectrum (**d**) for $\varepsilon_{r}=9.9$ and different $\sigma_{\mathrm{rms}}$ values.

The wake potential, transfer impedance, output signal voltage, and spectrum for the round BPM are shown in Figure 4, which is obtained by considering three different bunch lengths ($\sigma_{\mathrm{rms}}=3,\;5,\;10$ mm). In particular, these curves are derived by adopting alumina 99.5% for the vacuum sealing material ($\varepsilon_{r}=9.9$) and limiting the wake length to one meter in order to keep as short as possible but still meaningful for the numerical evaluation. The reliability of this latter choice is confirmed by the −80 dB decrease in the field energy over time achieved at the end of the simulation.

This first set of results shows that the shorter the bunch length, the higher the wake potential, its time duration, and the energy lost by the beam in the BPM structure. Note that, as the bunch length decreases, the corresponding beam coupling impedance plot covers, as expected, the calculated curves for longer bunches, while trapped modes not excited by long bunches appear as resonance peaks when the bunch spectrum increases. Additionally, the figure reveals that the output signal strength increases as the bunch length decreases, a behavior that could lead to very high electric fields inside the button gap in the case of short bunches.

**Figure 5:**
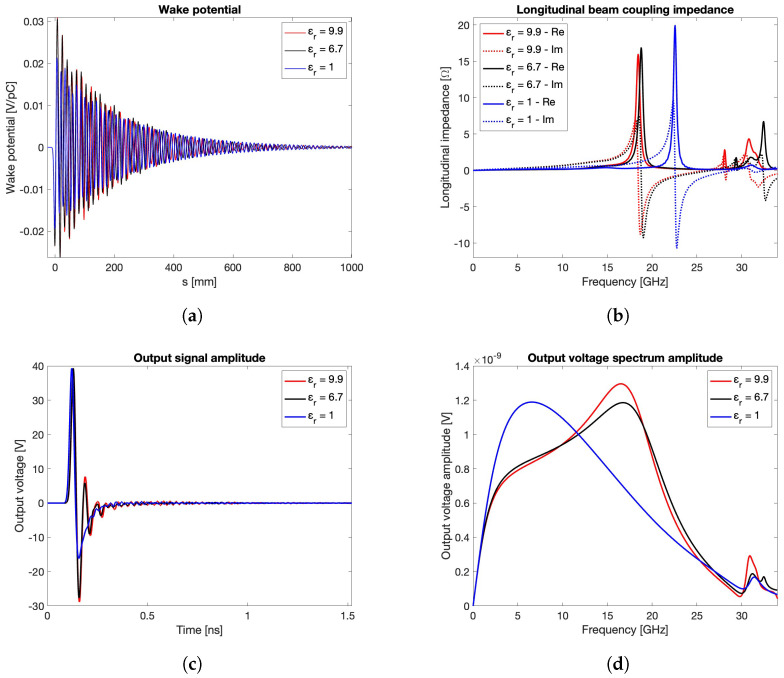
Wake potential (**a**), beam coupling impedance (**b**), output signal voltage (**c**), and spectrum (**d**) for $\sigma_{\mathrm{rms}}=3$ mm and different $\varepsilon_{r}$ values.

A second set of simulations is carried out by fixing the bunch length to $\sigma_{\mathrm{rms}}=3$ mm and considering, for the vacuum sealing material D, three different values of the relative dielectric permittivity $\varepsilon_{r}=1,\;6.7,\;9.9$. The derived curves are shown in Figure 5, which still reports the wake potentials, the transfer impedances, the output signal voltages, and their spectra. These results show how the beam coupling impedance resonant peak due to the first trapped mode shifts toward lower frequencies when $\varepsilon_{r}$ increases, as expected in terms of dielectric spectroscopy. Furthermore, the output signal shows a growing ringing due to reflections induced by the impedance mismatch along the PU extraction signal path.

**Figure 6:**
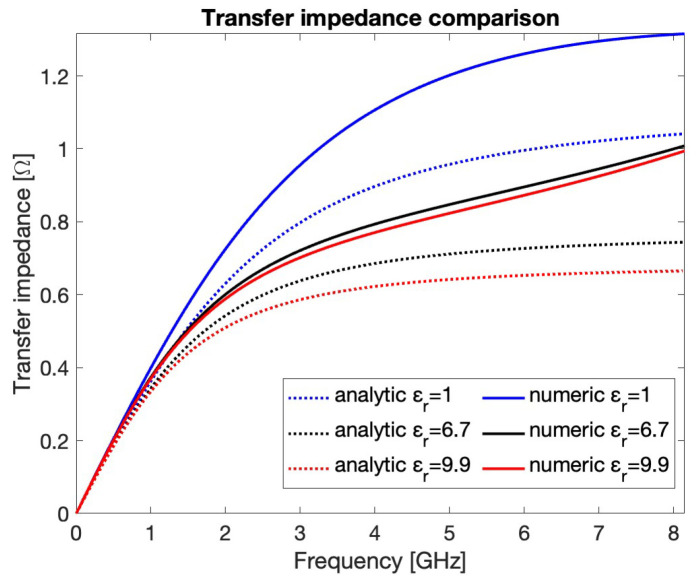
Analytical and numerical transfer impedance modulus for $\sigma_{\mathrm{rms}}=3$ mm and different $\varepsilon_{r}$ values.

In order to compare the transfer impedance calculated by the theoretical model with the one obtained by the numerical solver, the $C_{\mathrm{PU}}$ value must be estimated. To overcome the difficulties discussed at the end of Section 2.1 concerning the analytical derivation of this latter parameter, a set of electrostatic simulations has been carried out by adopting the CST electrostatic solver and varying the permittivity of the sealing material used in the PU. The obtained $C_{\mathrm{PU}}$ values are 1.11 pF, 1.61 pF, and 1.80 pF for $\varepsilon_{r}=1,\;6.7$, and 9.9, respectively. The corresponding $f_{\mathrm{cut}}$ are 2.87 GHz, 1.97 GHz, and 1.77 GHz. Figure 6 shows the comparison between the analytical (dotted lines) and numerical (solid lines) curves of the transfer impedance modulus for $\sigma_{\mathrm{rms}}=3$ mm. For $f\ll f_{\mathrm{cut}}$, the two models match very well. Due to the totally different approach between the two methods, the computational cost of the theoretical calculation is negligible compared to that of the numerical solver.

### 3.2. Square/Rhomboidal Pipes with Conical PUs

While the round pipe is often employed in many of the operating particle accelerators, alternative geometries relying on a rhomboidal structure have been adopted in other machines, such as Elettra and Elettra 2.0 storage rings [7]. Ther are two main reasons for this choice.

**Figure 7:**
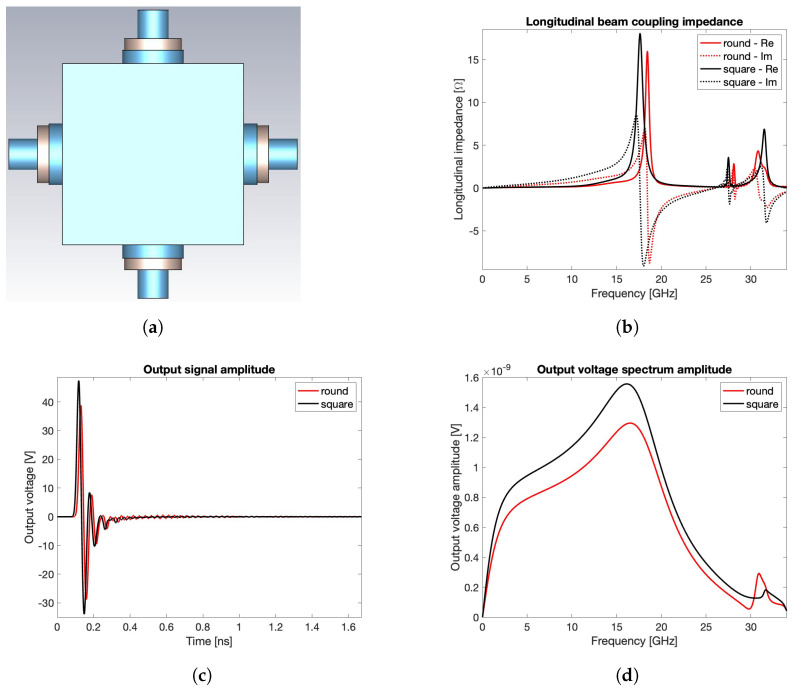
Comparison between round and square geometries: (**a**) square BPM model, (**b**) beam coupling impedance, (**c**) output signal voltage, and (**d**) spectrum.

The first reason is related to the manufacturing process, since a BPM body consisting of flat surfaces allows a simpler hosting of the buttons, without the need of leaving non-uniform gaps between the inner surface of the vacuum chamber and the buttons, a geometric configuration that might trigger undesired EM effects. The second, and more relevant reason, concerns the constraints imposed by other critical devices, such as magnetic element fitting, or other design considerations, such as X-ray-induced thermal loading effects. Regardless of the specific reason that imposes a non-circular vacuum pipe, the farther the BPM from a circular shape, the lower the reliability of the analytical model because of the lack of uniformity in the distribution of the induced surface charge and current. In this case, despite its higher computation cost, the numerical calculation method should always be used even if a raw estimation of the BPM transfer impedance is required. If the effective geometry of the BPM device can be, at a first evaluation, substituted by a purely square cross-section aligned with the coordinate planes, a more effective mesh coverage can be obtained by the CST wake field solver. This choice allows shorter calculation times for the preliminary investigation of the transfer impedances for different types of PU. To deepen this aspect, let us compare the round BPM with $r_{\mathrm{pipe}}=8.5$ mm (Figure 1b) and the square one with edge equal to 16 mm (Figure 7a), assuming $\sigma_{\mathrm{rms}}=3$ mm. The PU geometry is maintained cylindrical with sealing material having $\varepsilon_{r}=9.9$. The result of this comparison may be discussed by preliminarily observing the output voltages in Figure 4c and Figure 7c and the spectra in Figure 4d and Figure 7d, together with the beam coupling impedances in Figure 4b and Figure 7b. These figures reveal a good agreement between the respective performance figures corresponding to the two structures, further showing that the highest contribution to the beam coupling impedance is due to the non-propagating modes inside each of the four PUs. This implies that, between the two compared geometries, it might be preferable to select the one that guarantees a minor confinement of the higher order modes within the BPM body.

**Figure 8:**
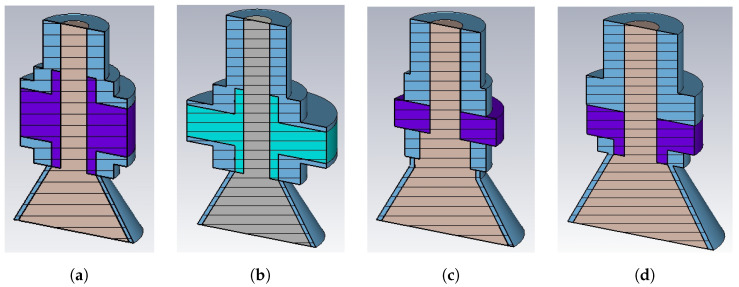
Compared PU shapes: (**a**) PU1, (**b**) PU2, (**c**) PU3, (**d**) PU4.

Beside alternative shapes for the vacuum chamber, accelerator physicists and engineers are also exploring non-cylindrical geometries for the electrode of the PU [23,24,25,26,27], with the purpose of matching the recent trend aimed to shift towards ever higher frequencies the peak of the real part of the beam coupling impedance. The results reported in the literature suggest that the conical shape is a good compromise between practical manufacturability and high frequency performance. However, similarly to the cylindrical shape, the impedance matching between the conical, transition, and coaxial PU sections strongly affects the PU energy reflection back into the vacuum pipe. To deepen this aspect, Figure 8 shows, for $\sigma_{\mathrm{rms}}=3$ mm and still considering a wake length limited to one meter, some of the PU geometries that have been investigated to find the best candidate to be used in Elettra 2.0. According to the classical formula for the characteristic impedance of the coaxial transmission lines, the coaxial section of the PUs have the ratio between the radii of the facing conductors equal to 2.3 to match the 50 Ω in air. The parameters used to compare the PUs are the loss factor $k_{∥}$, the ringing over time of the PU output signal, its spectrum, the time duration of the wake potential, and the beam coupling impedance. Due to the effect of the energy stored in the dielectric material used for the vacuum sealing, none of the PUs simulated has been able to fade the wake potential within 2 ns, that is, the radio frequency (RF) period of most synchrotrons. The losses are indicated in Table 1. All the PUs having a loss factor greater than 5 mV/pC have been excluded from further investigations. The remaining PUs were compared to find the best compromise between transfer impedance and beam coupling impedance with PU2 and PU4, which have been finally selected as possible candidates for real applications based on different types of vacuum sealing insulators.

**Figure 9:**
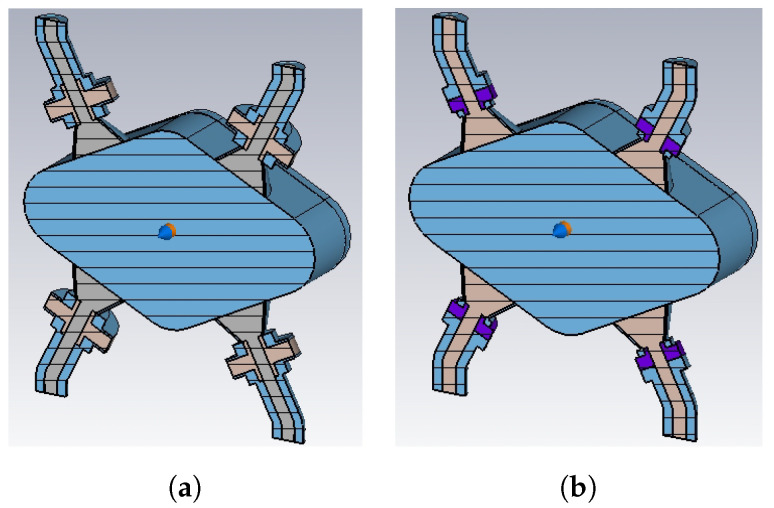
Rhomboidal BPM models based on PU2 (**a**) and PU4 (**b**).

**Figure 10:**
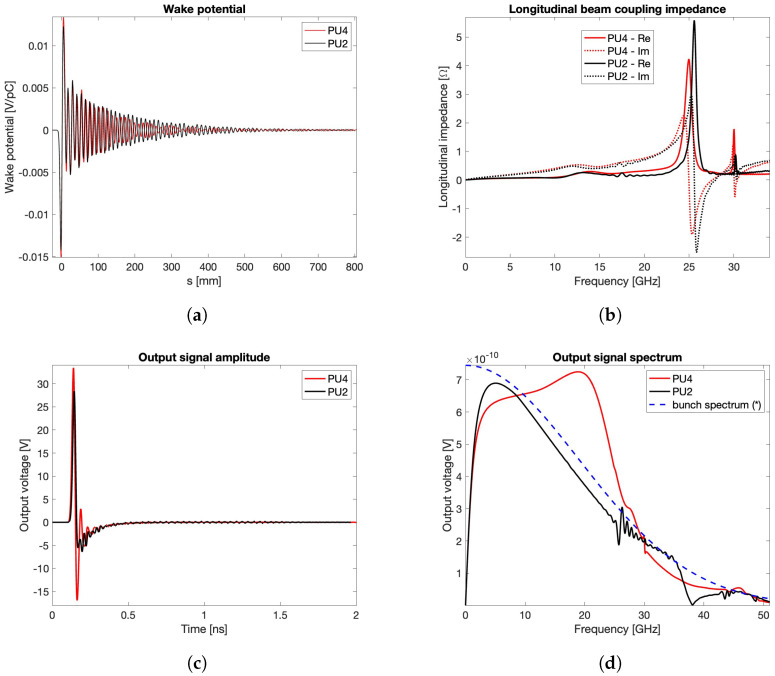
Wake potential (**a**), beam coupling impedance (**b**), output signal voltage (**c**), and spectrum (**d**) for $\sigma_{\mathrm{rms}}=3$ mm. (*): arbitrary unit.

Once the suitable PU shapes are selected, the final numerical step consists of considering the actual geometry of the vacuum chamber. As outlined at the beginning of this subsection and according to the decision taken for Elettra, even the fourth-generation Elettra 2.0 machine will have a rhomboidal BPM pipe, with a geometric scaling factor about three times smaller. The simulated BPMs based on PU2 and PU4 are reported in Figure 9, while the resulting wake potential, transfer impedance, output signal voltage, and spectrum are illustrated in Figure 10 ($\sigma_{\mathrm{rms}}=3$ mm). The dotted line shown in Figure 10d, representing the bunch spectrum rescaled to fit the vertical axis span, has been added in order to check the bandwidth overlapping of the PU signals with the one of the exciting beam.

## 4. Experimental Results

**Figure 11:**
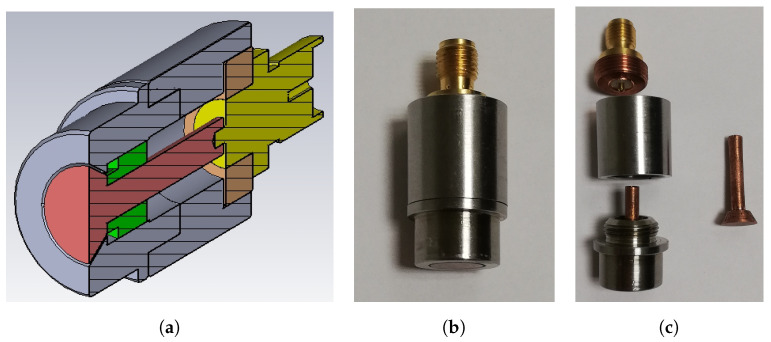
Manufactured PU: (**a**) cross-section, (**b**) assembled, (**c**) disassembled.

The experimental verification of the reliability of the numerical simulations has been carried out by two sets of measurements performed on a group of four (nominally identical) in-house manufactured PUs (Figure 11), which are based on the PU4 shape (Figure 8d). The used materials are copper for the central pin, ceramic for the vacuum sealing ($\varepsilon_{r}=6$), and stainless steel for the housing. The RF connector is a commercial SubMiniature version A (SMA) and the coaxial section is 50 Ω matched. Due to manufacturing tooling constraints, the size of these PUs is matched to the BPM body dimensions of Elettra, that is, bigger than the ones planned for Elettra 2.0. This is an acceptable oversize because even the RF simulations related to the experimental results have been performed on the geometrical size of the Elettra vacuum pipe.

### 4.1. TDR Measurements

**Figure 12:**
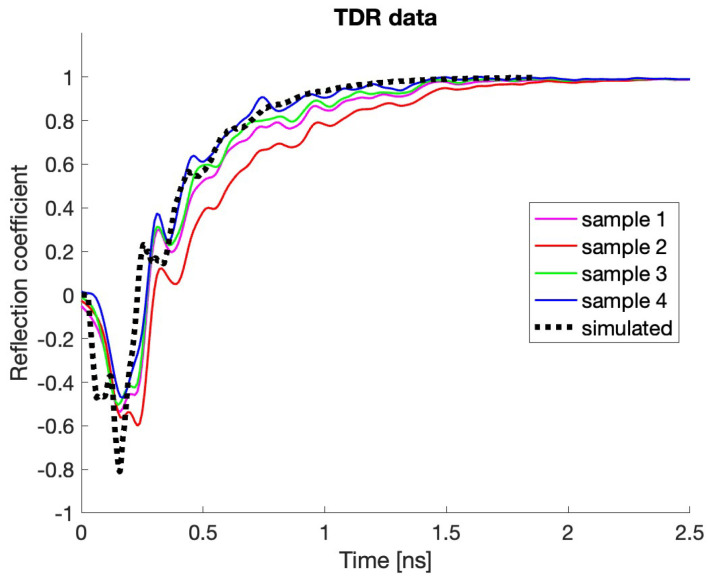
Experimental and simulated TDR signals for the manufactured PUs.

The first set of experimental values has been obtained by using a Teledyne T3SP15D reflectometer, which is employed to measure the TDR characteristics of the four manufactured PUs in open-circuit mode. The obtained results are reported in Figure 12, where the measured curves are represented by the four colored thin lines, while the simulated one is indicated by the black dotted line. Except from a moderate deviation of the red curve, the other three experimental ones are really close to the numerically derived performance, with the observed dispersion that may due to the manufacturing process, which, at this preliminary stage, has not been optimized.

### 4.2. VNA Measurements

**Figure 13:**
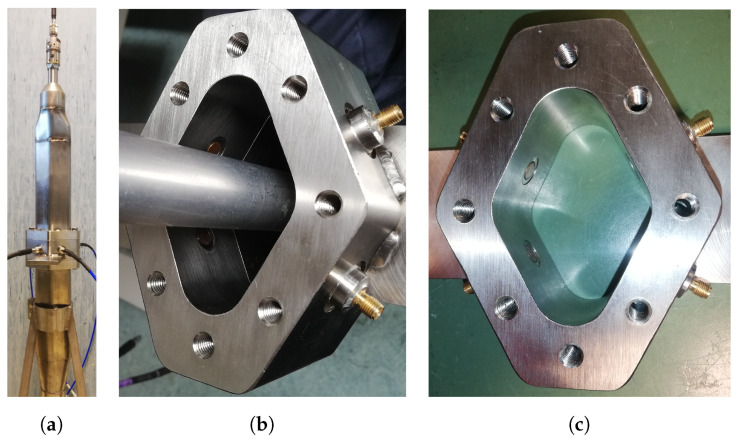
VNA measurements: (**a**) testbed, (**b**) inner coaxial condutor, (**c**) PU electrodes.

**Figure 14:**
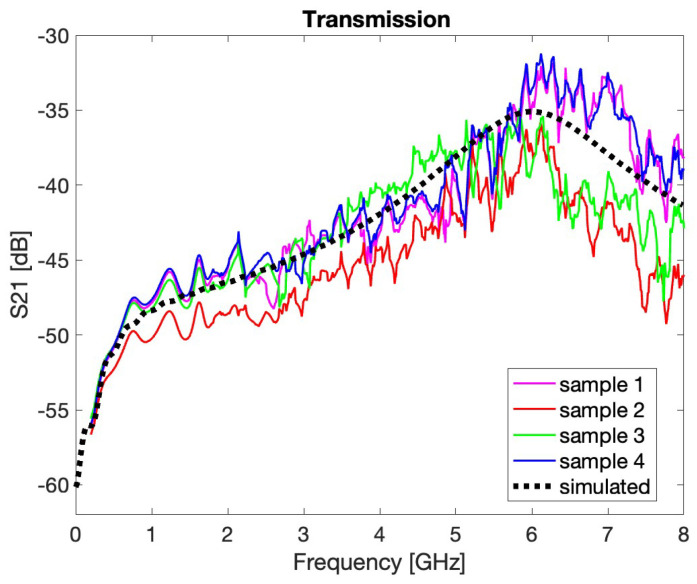
Experimental and simulated transmission coefficient for the manufactured PUs.

The second set of laboratory tests on the in-house PUs is carried out through a VNA to assess how the buttons couple to a particle beam as the frequency changes. The measurement setup consists of two sections of a vacuum chamber with the dimensions equal to those of the current Elettra accelerator, in which a BPM body can be inserted in a central position (Figure 13a). The two ends of the vacuum chamber are connected to a pair of N-connectors, which internally support an aluminum conductor of dimensions approximating a coaxial conductor adapted to 50 Ω impedance (Figure 13b). Through the dedicated ports, it is then possible to feed the system with an RF signal terminating on a matched load. The buttons in the central BPM couple with the EM field (Figure 13c). Hence, by analyzing the present signal, it becomes possible to measure the frequency response of the button inserted into its operating environment. Within this testbed, the acquisitions have been carried out through a Keysight PNA network analyzer, which has been used to feed the coaxial line while reading the signal present on one of the four buttons, thus showing the trend of the $s_{21}$ parameter as a function of the frequency (Figure 14). Since this kind of measurement may be generally affected by various perturbations, the operating procedure has been focused on a comparative evaluation between the response of the cylindrical button currently used for the Elettra BPM and that of the prototype of the conical button, which will be used for the BPM of the forthcoming Elettra 2.0 accelerator. The figure shows an acceptable qualitative agreement between the four experimental transmittances and the simulated one.

## 5. Conclusions

The design of a rhomboidal BPM device with conical PUs has been proposed by considering a detailed exploration of the EM behavior of the different components. Moving from a theoretical and numerical analysis of the conventional round BPM with cylindrical PUs, CST simulations has been carried out to identify the advantages of the vacuum chamber geometry selected for the operating Elettra synchrotron and for the forthcoming Elettra 2.0 one. Exhaustive investigations, involving different materials, bunch standard deviations, and PU structures have been carried out to identify the most suitable configuration. The results have identified a specific PU shape, for which four prototypes have been manufactured and subsequently characterized through TDR and VNA measurements. The developed device has provided a satisfactory performance in conjunction with a considerable manageability of the realization, thus making the designed element suitable for deployment in the next-generation Elettra 2.0 light source.

## Figures and Tables

**Table 1:** Loss factor for the PUs in Figure 8.

	PU1	PU2	PU3	PU4
$\varepsilon_{r}$	6.7	4.1	6.7	6.7
$k_{∥}$ [mV/pC]	4.40	3.90	5.06	4.67

## Data Availability

Data are contained within the article.

## Abbreviations

The following abbreviations are used in this manuscript:

3D	Three-dimensional
BPM	Beam position monitor
DC	Direct current
EM	Electromagnetic
FD	Frequency domain
HOM	High-order modes
PU	Pick-up
RF	Radio frequency
SMA	SubMiniature version A
TD	Time domain
TDR	TD reflectometer
TEM	Trasverse EM
VNA	Vector network analyzer
